# Association between frankfurt horizontal–referenced vertical position of the maxillary first molar and mandibular asymmetry: a cross-sectional study

**DOI:** 10.1007/s44445-026-00163-x

**Published:** 2026-03-28

**Authors:** Bekir Osmanov, Pavlo Burlakov, Andrii Kopchak

**Affiliations:** 1 Department of Maxillofacial Surgery, Bogomolets National Medical University, Kyiv, Ukraine; 2 Department of Orthodontics, Bogomolets National Medical University, Kyiv, Ukraine

**Keywords:** Mandibular asymmetry, Facial asymmetry, Maxillary 1st molar, Cephalometry, Frankfort horizontal plane, Three-dimensional analysis

## Abstract

**Purpose:**

Facial symmetry is a key determinant of aesthetic balance and functional harmony. Unilateral vertical discrepancies in the maxillary dentition, particularly side-to-side discrepancy in the FH-referenced vertical position of the maxillary first molar (U6), has been hypothesized to be associated with functional mandibular deviation and skeletal asymmetry. However, the relationship between localized dentoalveolar vertical discrepancy and mandibular morphology remains poorly understood.

**Methods:**

Eighty-seven patients aged 16–35 years with mandibular asymmetry ≥ 2 mm on MSCT, operationally defined as a side-to-side difference in total mandibular length (ΔCo-Go-Gn) ≥ 2 mm, were included. Three-dimensional cephalometric analysis was performed using ProPlan CMF (Materialise, Belgium). Four variants of the Frankfort horizontal (FH) plane (FH1-FH4) were constructed to evaluate the influence of reference-plane definition on the measurements. Mandibular asymmetry was quantified by differences in ramus length (Co-Go), body length (Go-Gn), and total side length (Co-Go-Gn). The primary independent variable was the vertical discrepancy between FH and U6 (ΔFH-U6). In addition, a control group of patients (n = 63) with mandibular asymmetry (ΔCo-Go-Gn) < 2 mm was included for between-group comparison of ΔFH-U6.

**Results:**

Weak positive correlations were observed between ΔFH-U6 and mandibular asymmetry indices, particularly ΔCo-Go and ΔCo-Go-Gn (Spearman’s ρ = 0.21–0.28). ΔFH-U6 differed across FH-plane definitions (Friedman test, *p* = 0.012), but correlation estimates were comparable across FH constructions. However, the observed correlations were small, and none of the primary correlations remained statistically significant after Benjamini–Hochberg false discovery rate (FDR) adjustment. Patients with mandibular asymmetry ≥ 2 mm showed a slightly greater ΔFH-U6 than controls with asymmetry < 2 mm (median [IQR]: 0.9 [0.45–1.75] mm vs 0.8 [0.2–1.3] mm; Mann–Whitney U test, *p* = 0.049).

**Conclusion:**

FH-referenced vertical discrepancy of the maxillary first molar may show a small exploratory association with mandibular asymmetry and was slightly greater in patients with clinically relevant mandibular asymmetry than in controls.

## Introduction

Facial symmetry is a crucial determinant of physical attractiveness from the perspectives of evolutionary biology, psychology, and aesthetics. Within the framework of evolutionary theory, symmetry is regarded as an indicator of genetic stability and efficient embryonic development, suggesting the absence of major mutations or the influence of exogenous factors during critical periods of morphogenesis (Harun et al. 2023; Obrochta et al. 2025). Studies have consistently demonstrated that symmetric faces are perceived as more attractive across different cultures, emphasizing the universality of this preference (Harun et al. 2023; Little et al. 2011). Although absolute facial symmetry is rare in humans, even mild asymmetry can affect the perception of appearance (Eißing et al. 2024). The issue of facial symmetry is particularly relevant in medical practice—especially in orthodontics and maxillofacial surgery—where the diagnosis and correction of asymmetry are essential for achieving both aesthetic and functional outcomes.

The causes of facial asymmetry may be either congenital or acquired. Congenital factors include genetic abnormalities affecting the development of the first and second branchial arches, exposure to teratogenic agents, intrauterine pressure, impaired blood supply, or disturbances in the migration of neural crest cells (Kessler et al. 2025; Thiesen et al. 2015). Acquired causes encompass trauma, infections, neoplasms, as well as functional disorders such as asymmetric masticatory load or malocclusion. Pathologies of the temporomandibular joint (TMJ)—including juvenile arthritis, condylar hyperplasia, and ankylosis—also play a significant role in the development of asymmetry (Kessler et al. 2025; Thiesen et al. 2015). The mandible plays a key role in the formation of facial asymmetry, as its shape and position largely determine overall facial balance. In particular, functional mandibular shifts have been discussed in relation to occlusal interferences and may contribute to asymmetric mandibular development (Bono 2025).

Against this background, increasing attention has been directed toward localized posterior dentoalveolar vertical discrepancies and their possible relationship with mandibular morphological asymmetry. In the present study, the maxillary first molar (U6) was used as a standardized posterior reference point for quantifying side-to-side vertical discrepancy. Such discrepancies and related functional deviations have been hypothesized to be associated with uneven loading of the temporomandibular joint and masticatory muscles, potentially affecting condylar, ramal, or mandibular body morphology (Miresmaeili et al. 2021; Tai et al. 2012; Zou et al. 2022). A similar mechanism has also been proposed as a potential etiological factor contributing to pronounced craniofacial asymmetries in patients with disturbances in molar eruption (Akbulut and Soğanci 2022; Kim et al. 2014). However, the precise relationship between side-to-side discrepancy of individual teeth—particularly U6—and mandibular morphological asymmetry remains insufficiently investigated.

Recent treatment-oriented studies have further emphasized the clinical relevance of cant-related asymmetry by showing that anterior unilateral bite turbos can be used to correct occlusal or incisal cant associated with asymmetric overbite (Viet Anh et al. 2025; Nguyen et al. 2025). These reports suggest that localized vertical discrepancies may be clinically actionable, even though their morphologic correlates remain insufficiently understood.

Posterior dentoalveolar discrepancies have also been discussed within the occlusal-plane concept developed by Sadao Sato and co-workers. According to this framework, alterations in the posterior occlusal plane may induce mandibular lateral displacement and contribute to the development of dentoskeletal asymmetry. In this context, vertical discrepancies in posterior tooth position relative to cranial reference planes may represent one of the morphological correlates of occlusal imbalance (Tanaka and Sato 2008; Ishizaki et al. 2010). Therefore, evaluating vertical posterior tooth position relative to a cranial reference plane such as the Frankfort horizontal may provide additional insight into potential occlusal contributions to mandibular asymmetry.

The aim of this study was to evaluate the association between side-to-side discrepancy in the FH-referenced vertical position of the maxillary first molar (U6) and mandibular asymmetry, assessed by ramus length (Co-Go), body length (Go-Gn), and total mandibular length (Co-Go-Gn), in patients with preexisting mandibular asymmetry ≥ 2 mm, and to compare ΔFH-U6 between these patients and a control group with mandibular asymmetry < 2 mm. The null hypothesis was that there is no association between the side-to-side difference in the FH-referenced vertical position of the maxillary first molar (ΔFH-U6) and mandibular morphological asymmetry parameters (ΔCo-Go, ΔGo-Gn, ΔCo-Go-Gn). An additional objective was to assess whether the severity of mandibular asymmetry is related to sagittal and vertical jaw relationships, mandibular growth pattern, as well as patient age and sex. Because the present study was limited to three-dimensional morphologic assessment, it did not directly evaluate functional shift, occlusal contact timing, chewing dominance, or TMJ status.

## Material and methods

This retrospective cross-sectional study included patients treated at Bogomolets National Medical University, Kyiv, Ukraine, between 2022 and 2025, who were referred for evaluation of malocclusion and suspected mandibular asymmetry. Clinical suspicion of mandibular asymmetry was based on routine clinical examination and included one or more of the following findings: deviation of the chin point, dental midline discrepancy, unilateral posterior crossbite, occlusal cant, and/or a functional mandibular shift on closure. Multislice computed tomography (MSCT) imaging was obtained as part of the diagnostic work-up and treatment planning to enable three-dimensional cephalometric assessment and was not performed solely for research purposes.

### Study groups and eligibility criteria

The study included two groups: an asymmetry group and a control group. Inclusion criteria for the asymmetry group were age 16–35 years, MSCT-confirmed mandibular asymmetry (ΔCo-Go-Gn) ≥ 2 mm and informed consent to participate in the study. Inclusion criteria for the control group were identical, except that mandibular asymmetry was < 2 mm.

Exclusion criteria for both groups comprised the presence of congenital craniofacial deformities, previous or ongoing orthodontic treatment, absence of adjacent teeth to the maxillary first molar (U5 and U7; FDI 15/17 or 25/27) or absence of the opposing mandibular first molar (L6; FDI 36/46). These exclusions were applied intentionally to preserve a more standardized posterior occlusal context for evaluating the association between U6 vertical discrepancy and mandibular asymmetry, rather than the effects of missing teeth or reduced posterior support. Patients with a history of facial skeletal trauma, condylar hyperplasia or hypoplasia, rheumatic diseases associated with temporomandibular joint (TMJ) condylar head resorption, significant carious lesions or restorations of U6, as well as those with MSCT images of insufficient quality (artifacts, incomplete coverage of the jaws or TMJs, or spatial misalignment) were also excluded. Additionally, patients with known neurological or neuromuscular disorders that could affect mandibular position or development were not included.

Of the 213 patients evaluated for alveolar or skeletal forms of malocclusion with clinically suspected mandibular asymmetry, 87 met the criteria for the asymmetry group (≥ 2 mm), and 63 met the criteria for the control group (< 2 mm).

### Outcomes and exposure

All detailed morphometric measurements, alternative FH-plane constructions, and correlation analyses were performed in the asymmetry group only. The control group was used exclusively for between-group comparison of ΔFH-U6 and was not included in the primary correlation analyses. For between-group comparison, ΔFH-U6 was calculated using FH1 as the primary reference-plane definition.

#### Primary outcomes

The primary outcomes were mandibular side-to-side asymmetry measures expressed as absolute differences in (1) ramus length (ΔCo-Go), (2) body length (ΔGo-Gn), and (3) total mandibular length (ΔCo-Go-Gn). Each outcome was analyzed separately; no composite asymmetry score was used.

#### Primary variables

**Fig. 1:**
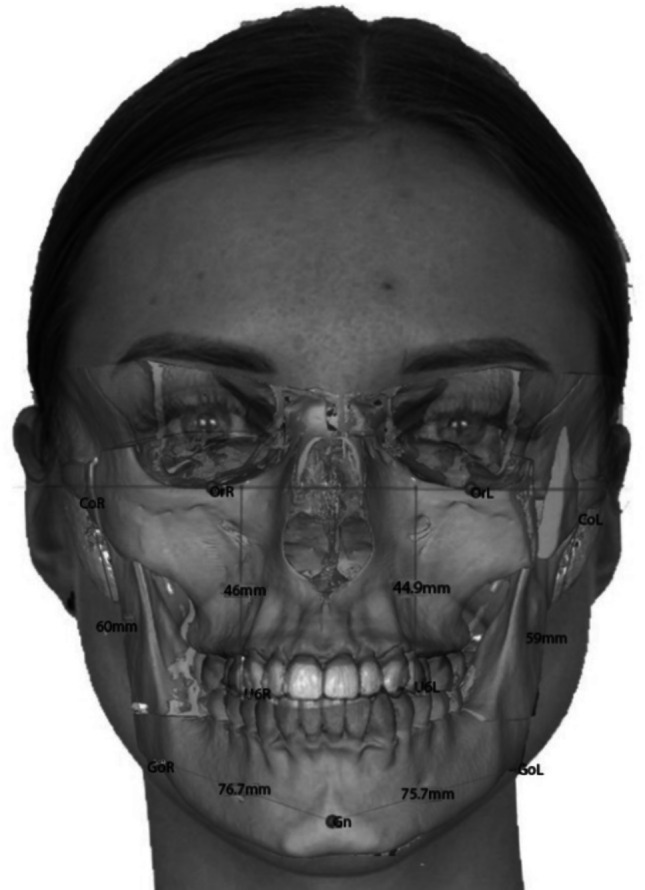
Definition of side-to-side discrepancy in the FH-referenced vertical position of the maxillary first molar (U6) illustrated by a clinical photograph superimposed on the CT-derived model, showing a higher vertical position of U6 on one side relative to the contralateral side in maximum intercuspation

The primary independent variable was the side-to-side difference in the FH-referenced vertical position of the maxillary first molar (U6). In this study, this variable was defined as the difference in the vertical position of the maxillary first molar (FDI 16/26) between the right and left sides relative to the Frankfort horizontal plane in centric occlusion (Fig. 1). The exposure variable in the present study represents a side-to-side difference in the FH-referenced vertical position of U6 and should not be interpreted as direct evidence of verified occlusal interference or premature occlusal contact.

The vertical position of U6 was quantified using the mesiopalatal cusp tip, selected as a single, consistently identifiable posterior landmark. This cusp represents the main supporting (functional) cusp of the maxillary first molar and typically forms stable contacts with the opposing mandibular dentition in maximum intercuspation; therefore, a vertical discrepancy at this cusp was considered more likely to be functionally relevant than discrepancies at less engaged posterior cusp landmarks. In contrast, buccal cusps primarily contribute to guiding/protective occlusal function, and the distopalatal cusp tip may be less distinctly visualized on CT-derived surface models, which can reduce landmark repeatability. Accordingly, one cusp-tip landmark was placed at the mesiopalatal cusp tip of each maxillary first molar clinical crown.

For each side, the perpendicular distance from the Frankfort horizontal plane (FH) to this cusp tip was measured to obtain the FH-U6 value. The side-to-side difference was calculated as ΔFH-U6 =|(FH-U6)_right − (FH-U6)_left| and was analyzed as a continuous variable.

The use of U6 in the present study was not intended to imply that the observed discrepancy was necessarily restricted to a single tooth; rather, U6 was selected as a standardized posterior landmark within the dentition for quantifying side-to-side vertical discrepancy.

#### Secondary variables

Secondary variables included sagittal skeletal relationships (SNA, SNB, ANB), vertical skeletal relationships (LFH/TFH ratio), and mandibular growth pattern (gonial angle), as well as categorical malocclusion characteristics (Angle Class I/II/III and vertical pattern groups). These variables were analyzed in an exploratory manner to assess their potential associations with the asymmetry outcomes (ΔCo-Go, ΔGo-Gn, ΔCo-Go-Gn). Additionally, the influence of patients’ sex and age on the degree of mandibular asymmetry was evaluated.

### Image acquisition and measurements

**Fig. 2:**
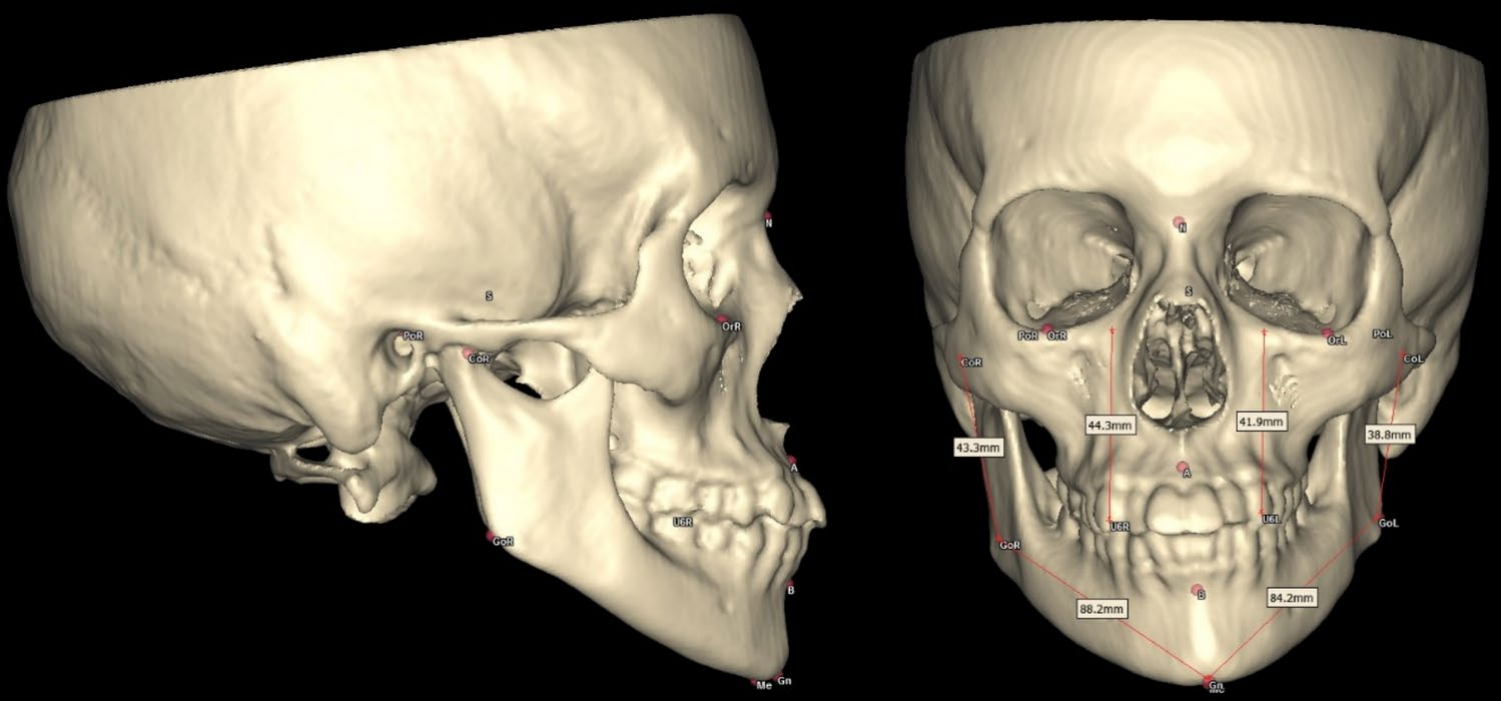
Cephalometric landmarks used for mandibular morphology and symmetry assessment. Three-dimensional reconstruction of the craniofacial skeleton based on MSCT data, shown in two projections. Landmarks used for mandibular morphology and symmetry assessment are indicated

All patients underwent MSCT as part of routine diagnostic work-up and treatment planning (not performed solely for research purposes). In our center, when facial asymmetry was clinically suspected, MSCT was used as part of the diagnostic pathway during the study period because it allowed a more comprehensive craniofacial assessment and provided better soft-tissue contrast than CBCT. In routine clinical practice, the imaging modality was selected to support comprehensive diagnostic needs beyond skeletal measurements (including potential soft-tissue assessment), even though the present study utilized only the hard-tissue component for 3D cephalometric analysis. Scans were obtained with the mandible in centric occlusion. During MSCT acquisition, patients were instructed to maintain their usual maximum intercuspation with the teeth in contact and the mandible in a relaxed, closed position. No additional bite registration device was used; head positioning followed the standard stabilization protocol of the imaging unit. DICOM datasets were exported and imported into ProPlan CMF (Materialise, Belgium) for segmentation, three-dimensional reconstruction, and cephalometric measurements. MSCT DICOM datasets were segmented using a bone threshold mask (226–3071 Hounsfield units), after which a 3D model of the craniofacial hard tissues was generated. This threshold range was used to isolate mineralized craniofacial structures and exclude soft tissues, following common CT-based craniofacial segmentation practice. Anatomical landmarks were identified on the reconstructed models to assess mandibular morphology and symmetry, as well as the geometry of the mandibular rami and body and intermaxillary relationships (Fig. 2). All anatomical landmarks used to assess mandibular morphology and symmetry are listed in Table 1. Definitions of all cephalometric measurement parameters—including Co-Go, Go-Gn, Co-Go-Gn, and the ΔFH-U6 distance—are presented in Table 2. These parameters formed the basis for all linear measurements and asymmetry calculations used in the present study.

**Table 1:** Cephalometric landmarks used in the study

Landmark name	Abbreviation	Definition
Sella turcica	S	The center of the sella turcica at the cranial base
Nasion	N	The point at the intersection of the midsagittal plane and the frontonasal suture
Anterior Nasal Spine	ANS	The most anterior point of the anterior nasal spine
Point A	A	The deepest point on the anterior contour of the maxillary alveolar process in the midsagittal plane
Point B	B	The deepest point on the anterior contour of the mandibular alveolar process in the midsagittal plane
Gnathion	Gn	The point located at the junction of the lower border of the mandible and the outer contour of the symphysis
Menton	Me	The most inferior point on the mandibular symphysis in the midsagittal plane
Orbitale	Or	The most inferior point on the infraorbital margin
Porion	Po	The point located at the uppermost margin of the external auditory meatus
Gonion	Go	The point at the apex of the angle formed by the posterior border of the mandibular ramus and the inferior border of the mandibular body
Articulare	Ar	The point of intersection between the inferior contour of the cranial base and the posterior border of the mandibular condylar process
Condylion	Co	The most superior point on the mandibular condyle
Upper first molar	U6	Mesiopalatal cusp tip of the maxillary first molar (U6)

**Table 2:** Three-dimensional cephalometric parameters (planes, angles, and linear distances) used in the study

Parameter	Definition	Reference value
FH Plane 1	Constructed through points OrR, OrL, and PoL	-
FH Plane 2	Constructed through points OrR, OrL, and PoR	-
FH Plane 3	Constructed through points PoR, PoL, and OrR	-
FH Plane 4	Constructed through points PoR, PoL, and OrL	-
SNA angle	Formed by points S–N-A; indicates the anteroposterior position of the maxilla relative to the cranial base	82 ± 3.7⁰
SNB angle	Formed by points S–N-B; indicates the anteroposterior position of the mandible relative to the cranial base	80 ± 3.8⁰
Gonial angle	Formed by points Ar-Go-Me; reflects the mandibular growth pattern	124 ± 5⁰
Right ramus length	Linear distance between CoR and GoR, representing the length of the right mandibular ramus	-
Left ramus length	Linear distance between CoL and GoL, representing the length of the left mandibular ramus	-
Right body part length	Linear distance between GoR and Gn, representing the length of the right half of the mandibular body	-
Left body part length	Linear distance between GoL and Gn, representing the length of the left half of the mandibular body	-
FH-U6L distance	Perpendicular distance from the Frankfort horizontal plane (FH) to the mesiopalatal cusp tip of the left maxillary first molar (U6L)	-
FH-U6R distance	Perpendicular distance from the Frankfort horizontal plane (FH) to the mesiopalatal cusp tip of the right maxillary first molar (U6R)	-
Total Facial Height (TFH)	Linear distance between N and Me, representing the total facial height	-
Lower Facial Height (LFH)	Linear distance between ANS and Me, representing the lower third of the face	-
LFH/TFH Ratio	Ratio of the lower to total facial height (ANS-Me/N-Me), used to assess vertical facial proportions	53–57%

### Reliability assessment

To assess measurement repeatability, intra- and inter-examiner reliability were evaluated for FH-U6 (mesiopalatal cusp tip) and mandibular linear measurements (Co-Go, Go-Gn, Co-Go-Gn). Two examiners independently repeated landmark placement and measurements in a random subset of 30 cases, with a 2-week interval between sessions. The 3D models were reloaded and landmarks were placed again from scratch. Examiners were blinded to previous results. Intra- and inter-examiner reliability for FH–U6 and mandibular linear measurements was excellent. The ICC values ranged from 0.912 to 0.995 for intra-examiner agreement and from 0.931 to 0.996 for inter-examiner agreement. The corresponding SEM values ranged from 0.241 to 1.519 mm for intra-examiner assessment and from 0.265 to 1.237 mm for inter-examiner assessment. The Dahlberg error ranged from 0.264 to 1.653 mm for intra-examiner agreement and from 0.268 to 1.253 mm for inter-examiner agreement, indicating high repeatability of landmark identification and measurement procedures.

### Frankfort horizontal plane variability

**Fig. 3:**
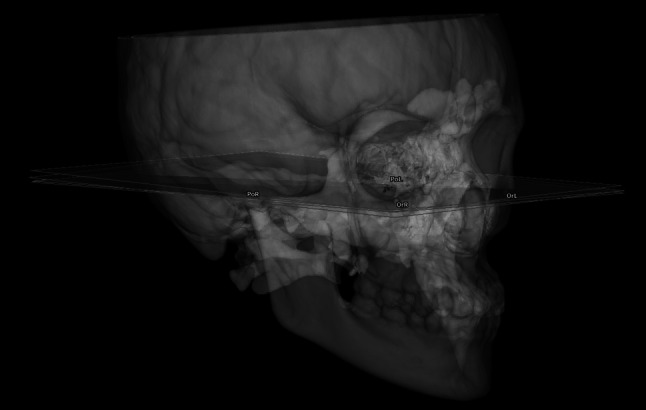
Alternative constructions of the Frankfort horizontal plane. Four alternative constructions of the Frankfort horizontal plane (FH1-FH4) based on different combinations of anatomical landmarks (OrL, OrR, PoL, PoR), illustrated on three-dimensional craniofacial models

Considering the well-documented variability in defining the Frankfort horizontal plane (FH) in three-dimensional cephalometry, four alternative constructions of the plane (FH1-FH4) were established in this study, each based on a different combination of three anatomical landmarks (OrL, OrR, PoL, PoR) (Fig. 3). For each variant, cephalometric measurements were performed separately, allowing for the assessment of the potential influence of landmark selection on the outcomes of the symmetry analysis (Mangal et al. 2020; Santos et al. 2017; Swennen et al. 2005).

### Statistical analysis

Data processing and statistical analyses were performed using the EZR software package (Easy R, version 1.61; Saitama Medical Center, Jichi Medical University, Japan) based on R version 4.2.2 (R Foundation for Statistical Computing, Vienna, Austria). The distribution of quantitative variables was assessed using the Shapiro–Wilk test. Because the data did not meet parametric assumptions, nonparametric methods were used throughout. Descriptive statistics are presented as medians (IQR) or means ± SD, as appropriate, and as counts with percentages. The Mann–Whitney U test was used for comparisons between two independent groups, the Kruskal–Wallis test for comparisons among three or more independent groups, and the Friedman test for comparisons among three or more paired (related) samples. Associations between morphological parameters were evaluated using Spearman’s rank correlation coefficient. Because multiple related comparisons were performed across FH-plane constructions and mandibular asymmetry outcomes, p-values for the 12 primary correlation tests were adjusted using the Benjamini–Hochberg false discovery rate procedure. For each Spearman correlation coefficient, 95% confidence intervals were estimated using bootstrap resampling. Both raw and adjusted p-values were reported. No multiplicity correction was applied to secondary subgroup analyses, which were interpreted as exploratory.

### Sample size determination

An a priori power analysis was performed in EZR (Easy R) using the pwr package (pwr.r.test) for a two-tailed correlation test (alpha = 0.05). Because prior data were unavailable, a medium effect size (|r|= 0.30) was assumed as the minimal clinically meaningful association. With power (1-beta) = 0.80, the required total sample size was n = 85. The final sample size (n = 87) met this requirement. The calculation is based on the bivariate normal correlation test and is used as an approximation for Spearman's rank correlation.

## Results

The asymmetry group comprised 87 patients with mandibular asymmetry ≥ 2 mm, and its descriptive characteristics are presented below. The control group comprised 63 patients with mandibular asymmetry < 2 mm and was used for between-group comparison of ΔFH-U6.

The mean age of the asymmetry group was 24.0 ± 5.9 years. The majority were females, accounting for 63.2% (n = 55) of the asymmetry group. The median difference in mandibular ramus length (Co-Go) between sides was 2.1 mm (IQR: 0.7–4.5 mm), while the median difference in mandibular body length (Go-Gn) was 2.4 mm (IQR: 1.2–3.5 mm). The overall side-to-side asymmetry of mandibular length (Co-Go-Gn) was more pronounced, with a median value of 4.0 mm (IQR: 2.8–5.6 mm).

Regarding sagittal relationships, most patients presented with Angle Class III malocclusion (35 patients, 40.2%), followed by Class II (32 patients, 36.7%) and Class I (20 patients, 22.9%). Mandibular ramus asymmetry was numerically higher in Class I patients (Median = 3.7 mm) than in Class II (Median = 1.9 mm) and Class III (Median = 1.6 mm); however, this difference did not reach statistical significance (Kruskal–Wallis test, *p* > 0.05; Table 3).

**Table 3:** Mandibular asymmetry measurements in various subgroups of the asymmetry group (≥ 2 mm)

Parameter	Sex*		Sagittal jaw relationship (Angle classification)**			Vertical facial proportions (LFH/TFH ratio) **			Mandibular growth pattern (gonial angle)**		
	M	F	Class I	Class II	Class III	Open-bite tendency (> 57%)	Normal (53–57%)	Deep-bite tendency (< 53%)	Vertical (< 117°)	Neutral (122 ± 5°)	Horizontal (> 127°)
N, %	32 (36.8%)	55 (63.2%)	20 (22.9%)	32 (36.7%)	35 (40.2%)	40 (46.0%)	40 (46.0%)	7 (8.0%)	33 (37.9%)	45 (51.7%)	9 (10.3%)
Ramus asymmetry (mm)	Median = 2.9 (IQR 0.9–5.3)	Median = 1.6 (IQR 0.7–4.1)	Median = 3.7 (IQR 0.7–5.75)	Median = 1.95 (IQR 0.8–3.65)	Median = 1.6 (IQR 0.7–4.5)	Median = 1.45 (IQR 0.55–4.1)	Median = 2.6 (IQR 1.4–3.6)	Median = 3.95 (IQR 2.6–5.65)	Median = 1.6 (IQR 0.9–4.1)	Median = 2.9 (IQR 0.9–4.5)	Median = 1.0 (IQR 0.4–4.8)
Body asymmetry (mm)	Median = 2.6 (IQR 1.2–3.5)	Median = 2.2 (IQR 1.3–3.9)	Median = 2.75 (IQR 1.2–3.7)	Median = 2.5 (IQR 1.05–4.15)	Median = 1.8 (IQR 1.2–3.5)	Median = 2.9 (IQR 1.1–4.5)	Median = 1.9 (IQR 1.0–3.35)	Median = 4.15 (IQR 3.0–5.6)	Median = 2.4 (IQR 1.4–3.5)	Median = 2.2 (IQR 1.0–3.6)	Median = 2.7 (IQR 1.4–3.2)
Total mandibular asymmetry (mm)	Median = 3.6 (IQR 2.6–5.5)	Median = 4.2 (IQR 3.15–6.3)	Median = 4.45 (IQR 3.4–6.15)	Median = 3.15 (IQR 2.6–5.05)	Median = 4.0 (IQR 2.9–5.7)	Median = 2.6 (IQR 0.7–7.3)	Median = 2.80 (IQR 1.4–4.5)	Median = 3.4 (IQR 2.5–3.9)	Median = 4.0 (IQR 3.0–5.7)	Median = 4.2 (IQR 2.8–5.5)	Median = 3.3 (IQR 2.5–3.8)

* Mann–Whitney U test (*p* > 0.05)

** Kruskal–Wallis test (*p* > 0.05)

Most patients with pronounced asymmetries exhibited a neutral mandibular growth pattern (45 patients, 51.7%). Based on the LFH/TFH ratio, vertical facial pattern was distributed as follows: open-bite tendency, 40 patients (46.0%); normal vertical proportions, 40 patients (46.0%); and reduced vertical facial proportions (deep-bite tendency), 7 patients (8.0%). In the reduced vertical proportions group, the median ramus length asymmetry was 3.95 mm, compared with 1.45 mm in the open-bite group and 2.6 mm in the normal group. Similarly, the median mandibular body length asymmetry was 4.15 mm in the reduced vertical proportions group, versus 2.9 mm in the open-bite group and 1.9 mm in the normal group. However, these between-group differences did not reach statistical significance (Kruskal–Wallis test, *p* > 0.05; Table 3).

Weak positive correlations were observed between side-to-side discrepancy in the FH-referenced vertical position of the maxillary first molar (U6), quantified as ΔFH-U6, and mandibular asymmetry parameters across different constructions of the Frankfort horizontal (FH) plane (Table 4).

**Table 4:** Differences across FH plane definitions indicate methodological sensitivity of ΔFH-U6 to the reference plane

ΔFH-U6R/L	FH1	FH2	FH3	FH4	p-value
Median (mm)	1.0	0.9	1.0	1.0	0.012*
Interquartile range (mm)	0.4–1.7	0.4–1.7	0.4–1.8	0.4–1.8	

*Friedman test

Importantly, this result represents a methodological effect: ΔFH-U6 values vary systematically depending on the FH plane definition used for the same subjects, indicating reference-plane-dependent measurement sensitivity, not biological variability.

ΔFH-U6 showed a small but statistically significant difference across FH-plane constructions (Friedman test, *p* = 0.012). Across the sample, ΔFH-U6 ranged from 0.0 to 3.4 mm under FH1 and FH2, and from 0.0 to 4.9 mm under FH3 and FH4. Despite this difference, Spearman correlation coefficients between ΔFH-U6 and mandibular asymmetry parameters remained consistently low (approximately ρ = 0.2–0.28) regardless of FH-plane definition. Several correlations reached nominal significance (raw *p* < 0.05); however, after FDR adjustment for the 12 primary tests, none remained statistically significant. Effect sizes were small and 95% confidence intervals were wide (Table 5).

**Table 5:** Spearman’s correlations between ΔFH-U6 and mandibular asymmetry parameters across FH-plane constructions

FH variant	Outcome	Spearman ρ	95% CI	p raw	p FDR
FH1	ΔRamus length (ΔCo-Go)	0.217	−0.010 to 0.429	0.049	0.113
FH1	ΔBody length (ΔGo-Gn)	−0.167	−0.374 to 0.048	0.130	0.157
FH1	ΔMandibular side length (ΔCo-Go-Gn)	0.216	0.003 to 0.417	0.050	0.113
FH2	ΔRamus length (ΔCo-Go)	0.247	0.023 to 0.444	0.025	0.098
FH2	ΔBody length (ΔGo-Gn)	−0.186	−0.391 to 0.039	0.092	0.122
FH2	ΔMandibular side length (ΔCo-Go-Gn)	0.207	−0.007 to 0.410	0.060	0.113
FH3	ΔRamus length (ΔCo-Go)	0.196	−0.024 to 0.390	0.076	0.113
FH3	ΔBody length (ΔGo-Gn)	0.023	−0.188 to 0.235	0.838	0.914
FH3	ΔMandibular side length (ΔCo-Go-Gn)	0.284	0.066 to 0.474	0.009	0.078
FH4	ΔRamus length (ΔCo-Go)	0.200	−0.015 to 0.398	0.071	0.113
FH4	ΔBody length (ΔGo-Gn)	0.007	−0.215 to 0.223	0.951	0.951
FH4	ΔMandibular side length (ΔCo-Go-Gn)	0.271	0.055 to 0.471	0.013	0.078

In the between-group comparison, patients with mandibular asymmetry ≥ 2 mm showed a slightly greater ΔFH-U6 than controls with asymmetry < 2 mm (median [IQR]: 0.9 [0.45–1.75] mm vs 0.8 [0.2–1.3] mm; Mann–Whitney U test, *p* = 0.049).

The potential influence of vertical and sagittal occlusal types, as well as the mandibular growth pattern (gonial angle), was additionally analyzed. No statistically significant associations were found between these parameters and mandibular asymmetry in this sample (*p* > 0.05); these exploratory analyses may have been underpowered. As expected, the LFH/TFH ratio correlated strongly with the gonial angle (ρ = 0.89, *p* < 0.0001), reflecting the typical morphology associated with an open-bite tendency.

The sagittal relationship (Angle Class I, II, or III) did not show statistically significant associations in this sample: correlations between sagittal class and the parameters ΔRamus length (ΔCo-Go), ΔBody length (ΔGo-Gn), and ΔMandibular side length (ΔCo-Go-Gn) did not exceed ρ = 0.14. Similarly, the gonial angle demonstrated no significant association with any of the mandibular asymmetry parameters (ρ < 0.10), indicating its relative independence within this sample.

In addition to the primary correlations, potential associations between mandibular morphological asymmetry parameters and general patient characteristics—such as age and sex were analyzed. The correlation analysis revealed no statistically significant relationships between age and asymmetry (*p* > 0.05). For instance, the correlation coefficient between age and ΔRamus length (ΔCo-Go) was ρ =  − 0.17, indicating a weak negative association. Similarly, correlations between age and ΔMandibular side length (ΔCo-Go-Gn) (ρ =  − 0.11) or ΔBody length (ΔGo-Gn) (ρ = 0.035) were minimal.

The highest, albeit still weak, correlation was observed between sex and ΔRamus length (ΔCo-Go) (ρ = 0.13), whereas other parameters—including ΔBody length (ΔGo-Gn) (ρ = 0.01) and ΔFH-U6 distance (ρ =  − 0.03)—showed no meaningful relationships.

Overall, the results of this study suggest a small association between side-to-side discrepancy in the FH-referenced vertical position of the maxillary first molar (U6) and mandibular asymmetry parameters, particularly ramus and total mandibular length differences. However, none of the primary correlations remained statistically significant after FDR adjustment, and the corresponding 95% confidence intervals were wide. These findings should therefore be interpreted as exploratory and of uncertain clinical impact.

## Discussion

The obtained results suggest that a side-to-side discrepancy in the FH-referenced vertical position of the maxillary first molar (U6) may be weakly associated with mandibular morphological asymmetry, particularly in the ramus region. Importantly, after FDR adjustment for the 12 primary tests, none of the observed correlations remained statistically significant, and the effect sizes were small, indicating limited precision and uncertain clinical impact. Other examined factors—such as occlusal type and mandibular growth pattern—did not show statistically significant associations within the studied sample. The inclusion of a control group allowed us to extend the analysis beyond co-variation within an asymmetry-selected cohort: patients with clinically relevant mandibular asymmetry showed a slightly greater ΔFH-U6 than controls; however, the between-group difference was small and does not support causal inference. Accordingly, any mechanistic or clinical interpretations related to occlusal interference or functional mandibular shift should be considered hypothesis-generating rather than demonstrated outcomes of the present study.

In two-dimensional cephalometric analysis (lateral cephalograms), the Frankfort horizontal (FH) plane is defined as the line connecting the *Orbitale* and *Porion* points. In three-dimensional cephalometry, however, according to classical geometry, both *Orbitale* and *Porion* points can lie on a single plane only if they are perfectly symmetrical relative to the midsagittal plane—a condition that rarely occurs in nature. Consequently, the FH plane in 3D space is defined by three points (Mangal et al. 2020; Santos et al. 2017; Swennen et al. 2005), which allows for several possible constructions: (1) OrR-PoR-PoL, (2) OrL-PoR-PoL, (3) OrL-OrR-PoL, and (4) OrL-OrR-PoR (Mangal et al. 2020). An alternative approach involves using averaged coordinates between the right and left landmarks (Swennen et al. 2005; Lin et al. 2015). Such differences in defining the FH plane may affect the outcomes of cephalometric analysis—even in patients without evident asymmetry (Mangal et al. 2020; Santos et al. 2017; Swennen et al. 2005). To account for this variability, all key measurements and analyses were repeated using the four FH constructions (FH1-FH4) previously described in the literature (Mangal et al. 2020). In our sample, ΔFH-U6 showed a small but statistically significant difference across FH variants (Friedman test, *p* = 0.012), underscoring reference-plane dependence of the metric; therefore, this component should be interpreted as a sensitivity analysis. While ΔFH-U6 is reference-plane dependent, the (weak) association pattern between ΔFH-U6 and mandibular asymmetry parameters remained broadly comparable across FH constructions and thus was not unique to a single FH definition. Nevertheless, transparent reporting and methodological standardization of FH construction remain important in three-dimensional cephalometric studies. A related limitation is that FH itself may be unstable in asymmetric skulls; therefore, ΔFH-U6 may partially reflect cranial-base landmark asymmetry rather than tooth position alone. Alternative reference strategies, such as averaged bilateral landmarks, cranial-base registration, or natural head position-based planes, should be evaluated in future studies.

To standardize the U6 vertical-position metric, we used the mesiopalatal cusp tip as a single, consistently identifiable posterior landmark. From a methodological perspective, we intentionally operationalized posterior vertical discrepancy using a single-tooth, FH-referenced landmark rather than constructing a posterior occlusal plane (POP) from multiple posterior teeth. First, within the plane-definition workflow used in our software, plane construction is limited to three reference points, whereas POP definitions typically require four or more posterior tooth points. Second, even when multi-point planes are constructed manually, they represent an approximation, because posterior teeth are inherently non-coplanar due to normal occlusal curvature (e.g., the Curve of Spee). Using multiple posterior points may therefore introduce additional variability through plane fitting/orientation and superimposition error. To minimize method-driven error and improve reproducibility, we selected a single posterior landmark referenced to FH, while acknowledging that adjacent posterior teeth and the entire posterior segment were not assessed and that a broader posterior dentoalveolar vertical discrepancy cannot be excluded (Francisco et al. 2025; Hwang et al. 2014).

In a broader clinical context, our findings align with reports linking unilateral posterior dentoalveolar disturbances to mandibular asymmetry. Akbulut and Soğanci (2022) demonstrated asymmetry of the mandibular ramus and condylar process in individuals with unilateral eruption disturbances of the second molars. Similarly, Kim et al. (2014) reported that vertical dentoalveolar discrepancies may contribute to facial asymmetry by limiting eruption space and altering tooth inclination. At the same time, other authors have offered alternative perspectives based on different clinical exposures. For instance, Surme et al. (2023) found no significant mandibular asymmetry following unilateral extraction of the first molar, and similar conclusions were reported by Thiesen et al. (2016), supporting the view that craniofacial asymmetry is multifactorial. Although these studies address unilateral dental alterations and asymmetric occlusal conditions, they are not directly comparable to our exposure (FH-referenced U6 vertical discrepancy) and are cited here to provide broader clinical context rather than as direct support or refutation of our findings.

Importantly, in our study, no significant associations were found between the degree of mandibular asymmetry and vertical or sagittal malocclusion patterns. Specifically, vertical discrepancies (open or deep bite), anteroposterior relationships (Angle Classes I, II, and III), and mandibular growth pattern (gonial angle) did not demonstrate statistically significant correlations with morphological asymmetry indices. A similar absence of association between vertical growth type and mandibular asymmetry was reported by other authors (Habib et al. 2022). However, given the weak effect sizes and the lack of FDR-significant correlations in our primary analyses, any interpretation that localized posterior vertical discrepancy may be more closely associated with mandibular asymmetry than broader malocclusion categories should be considered exploratory. Recent treatment-focused studies have shown that anterior unilateral bite turbos may be effective for correcting occlusal or incisal cant associated with asymmetric overbite (Viet Anh et al. 2025; Nguyen et al. 2025). Nevertheless, the findings of Siddiqui et al. (2024) emphasized that vertical mandibular asymmetry, especially in condylar height, in patients with unilateral distal occlusion (Angle’s Class II subdivision) may be related to the position of the maxillary first molar (U6).

Several mechanisms have been proposed in the literature (e.g., functional shifts, asymmetric loading, muscle adaptation) (Miresmaeili et al. 2021; Tai et al. 2012), but they were not evaluated in the present study and should therefore be viewed as hypotheses for future research. Experimental studies report that prolonged functional deviation may be associated with TMJ changes (e.g., condylar remodeling and cartilage alterations) (Zou et al. 2022; Ren et al. 2025; Liu et al. 2007; Noh et al. 2021). Because TMJ morphology was not assessed here, these mechanistic links cannot be used to explain our findings and remain speculative. Unilateral masticatory dominance has also been proposed as a contributor to asymmetric mechanical loading of the masticatory system (Umehara et al. 2025; Kwon et al. 2007; Guan et al. 2023). Similarly, experimental models indicate that functional mandibular deviation may be accompanied by adaptive changes in masticatory muscles; since these factors were not assessed in the present study, this interpretation should likewise be considered hypothesis-generating.

A related conceptual perspective was advanced by Sato and colleagues, who described mandibular lateral displacement as a possible adaptive response to posterior occlusal imbalance (Tanaka and Sato 2008; Ishizaki et al. 2010). Although the cross-sectional design of the present study precludes causal inference, the current findings may be discussed within this broader interpretative framework.

### Study limitations

Several limitations should be acknowledged when interpreting the present findings. First, the study was based on retrospectively selected, clinically indicated MSCT scans rather than a standardized research imaging protocol; therefore, selection bias related to imaging indication cannot be fully excluded. In addition, maximum intercuspation was maintained according to patient instruction during clinically acquired scans rather than a dedicated research occlusal protocol, and small mandibular position variations during imaging cannot be ruled out. Because no direct functional occlusal assessment (e.g., T-scan or articulating analysis) was performed, the observed FH-referenced vertical discrepancy should not be interpreted as confirmed premature contact.

Second, due to the cross-sectional design, temporality, causality, and directionality cannot be inferred (FH-referenced vertical discrepancy → asymmetry vs asymmetry → discrepancy vs shared underlying factors). Moreover, because absolute side-to-side differences were analyzed, the present study quantified the magnitude, but not the direction, of asymmetry; therefore, laterality concordance between U6 vertical discrepancy and mandibular asymmetry could not be assessed.

Third, generalizability is limited by sample characteristics. The included age range (16–35 years) spans late adolescence and adulthood; residual effects of ongoing craniofacial growth in the youngest participants cannot be fully excluded, and skeletal maturity was not directly assessed. Although age was explored as a covariate and showed no statistically significant associations with mandibular asymmetry parameters in this sample (*p* > 0.05), growth-related effects remain a potential limitation. In addition, we restricted the analysis to patients with intact posterior dentition to ensure internal consistency for the U6-focused exposure definition; consequently, the findings may not be generalizable to patients with missing posterior teeth, and no inference can be made for such cases.

Fourth, the exposure definition was intentionally limited to a single landmark on each U6. Because adjacent posterior teeth and the entire posterior segment were not assessed, the study cannot determine whether the observed asymmetry was truly isolated to U6 or represented a broader posterior dentoalveolar vertical discrepancy; likewise, potential buccal-segment contributions to posterior canting could not be evaluated. ΔFH-U6 also showed reference-plane dependence across FH constructions; therefore, the multi-FH approach should be viewed as a sensitivity analysis, and transparent reporting and standardization of reference-plane construction remain important in three-dimensional cephalometry.

Fifth, the observed associations between ΔFH-U6 and mandibular morphology were small, consistent with the multifactorial nature of mandibular asymmetry. After FDR adjustment for the 12 primary tests, none of the correlations remained statistically significant, and 95% confidence intervals were wide, indicating limited precision and uncertain clinical impact. Subgroup analyses were exploratory and may have been underpowered; because the sample size was planned for a larger effect size than those actually observed, non-significant subgroup findings should not be interpreted as evidence of no association.

Finally, not all forms of asymmetry can be attributed to functional or dentoalveolar disturbances and some may have a congenital origin. Soft-tissue factors, postural habits, and muscular tone were not assessed. Further studies incorporating genetic factors, masticatory dominance, functional mandibular shift, and muscle function are warranted to better understand mechanisms potentially underlying craniofacial asymmetry. In addition to the within-group associations, ΔFH-U6 was slightly greater in patients with clinically relevant mandibular asymmetry than in controls; however, these findings remain cross-sectional and exploratory and should not be interpreted causally.

## Conclusion

In this cross-sectional study, we observed weak, exploratory correlations between side-to-side discrepancy in the FH-referenced vertical position of the maxillary first molar (ΔFH-U6) and mandibular morphological asymmetry, most notably in the ramus region. The between-group difference versus controls with mandibular asymmetry < 2 mm was small, and after FDR adjustment none of the primary correlation tests remained statistically significant. Accordingly, ΔFH-U6 should be interpreted as an FH-referenced geometric measurement that is dependent on FH plane definition, rather than as direct evidence of occlusal interference or functional mandibular shift. Further studies using standardized reference-plane definitions and designs that can assess temporality, ideally with direct functional occlusal evaluation, are needed to clarify interpretation and potential clinical relevance.

## Data Availability

Data supporting the findings of this study are available from the corresponding author (BO) upon reasonable request.
